# Malacoplakia of the bladder combined with infected renal calculi: A case report

**DOI:** 10.3389/fsurg.2023.1044963

**Published:** 2023-01-26

**Authors:** Qunjun He, Yi Wang, Xian Chen, Baopiao Xia, Xuqiang Zeng, Binhui Wang, Yong Fang, Shulin Liu, Wujun Xu

**Affiliations:** ^1^ Department of Total Quality Management and Information Statistics, The Second Affiliated Hospital, Hengyang Medical School, University of South China, Hengyang, China; ^2^ Department of Urology, The Second Affiliated Hospital, Hengyang Medical School, University of South China, Hengyang, China; ^3^ Department of Pathology, The Second Affiliated Hospital, Hengyang Medical School, University of South China, Hengyang, China; ^4^ Department of Radiology, The Second Affiliated Hospital, Hengyang Medical School, University of South China, Hengyang, China

**Keywords:** malacoplakia of the bladder, transurethral resection, urinary tract infection, renal calculi, case report, renal calculi

## Abstract

**Introduction:**

The malacoplakia of the bladder is a rare chronic acquired infection- associated granulomatous disease and even less common in combination with urinary stones.

**Case Presentation:**

We report the case of a 58-year-old female patient with malacoplakia of the bladder combined with renal calculi. The patient was admitted to the hospital with bilateral low back pain for one month and space-occupying lesions of the bladder for three days. Preoperative imaging suggested space-occupying lesions of the bladder: high probability of bladder cancer. Following the anti-infection treatment, the transurethral electrodesiccation was performed on the space-occupying lesions of the bladder. Pathological examination confirmed the diagnosis of malacoplakia of the bladder. Left-sided percutaneous nephrolithotomy was performed electively to remove the predisposing factors of infection. After the operation, the patient continued to receive anti-infection treatment for two months. The patient had a good prognosis in the six-month follow-up.

**Conclusions:**

Malacoplakia of the bladder is easily misdiagnosed as bladder cancer before operation, and the diagnosis depends on pathological diagnosis. Complete removal of urinary calculi, infection and other inducing factors, is beneficial to the treatment of malacoplakia of the bladder.

## Introduction

Malacoplakia, a rare chronic acquired infection-associated granulomatous disease, also known as Von Hansemann's disease, was first reported by Michaelis and Gutmann in 1902 (1). Malacoplakia may affect any organ but frequently occurs in the genitourinary system, especially the bladder (2). Malacoplakia usually presents as a solitary tan plaque or nodule that can be easily confused with bladder tumors (3), which must rely on pathologic examination for a final diagnosis. At present, only a few cases of malacoplakia combined with urinary stones have been reported. We report here a case of malacoplakia of the bladder combined with a stone in the left infrarenal calyx and reviewed relevant literature.

## Patient information

A female patient, 58 years old, farmer, married, menopausal. Bilateral low back pain for more than 1 month, and space-occupying lesions of bladder found for 3 days. The patient had pain at the end of urination with interrupted urination for 6 months. Two years ago, both kidney stones were removed by right nephrectomy and left percutaneous nephrolithotomy, and the patient had a history of hypertension for 6 months.

## Clinical findings

T: 36.5, P: 59 beats/min, R: 18 beats/min, BP: 110/77 mmHg (maintained with antihypertensive medication). The patient's abdomen was flat and soft, with a 6-cm surgical scar on the right lumbar region (incisional lithotomy) and a 1-cm surgical scar on the left lumbar region (percutaneous nephrolithotomy). There was no percussion pain in both kidney and ureteral stroke pressure, and no abnormality of external genitalia was observed.

## Timeline

April 19, 2021: Bilateral low back pain for more than 1 month; Bladder occupancy was found for 3 days; Patient was admitted to hospital. The urinalysis test was 3+ leukocytes and positive for nitrites. CT examination of the whole abdomen suggested multiple stones in both kidneys complicated with mild hydronephrosis. The space- occupying lesions of the bladder were detected, and bladder cancer was considered.

April 21, 2021: MRI showed the space-occupying lesions of bladder neck, high probability of bladder cancer (grade:VI-RADS2).

April 22, 2021: Urine culture showed ESBL-producing *Escherichia coli*. Given 4.5 piperacillin (IV, q8 h) preoperatively for anti-infection for 1 week, and the recheck revealed that nitrite was converted to negative in the urinary routine.

April 27, 2021: The transurethral bladder mass electrosurgery was conducted under combined spinal and epidural analgesia.

April 29, 2021: Pathological diagnosis of specimen after electrodesiccation: Bladder malacoplakia. Immunohistochemistry: CK-pan (surface epithelial+), GATA-3 (surface epithelial+), CD68 (histiocytes+) Ki67 (+).

May 6, 2021: The ultrasound-guided left percutaneous nephrolithotomy (PCNL) was performed under general anesthesia, and the postoperative kidney-ureter-bladder (KUB) showed that the stone was removed.

May 15, 2021: The patient was discharged from the hospital.

June 15, 2021: Remove the DJ tube.

November 15, 2021: Cystoscopy showed that the mass disappeared and the mucosa recovered well.

## Diagnostic assessment

**Figure 1:**
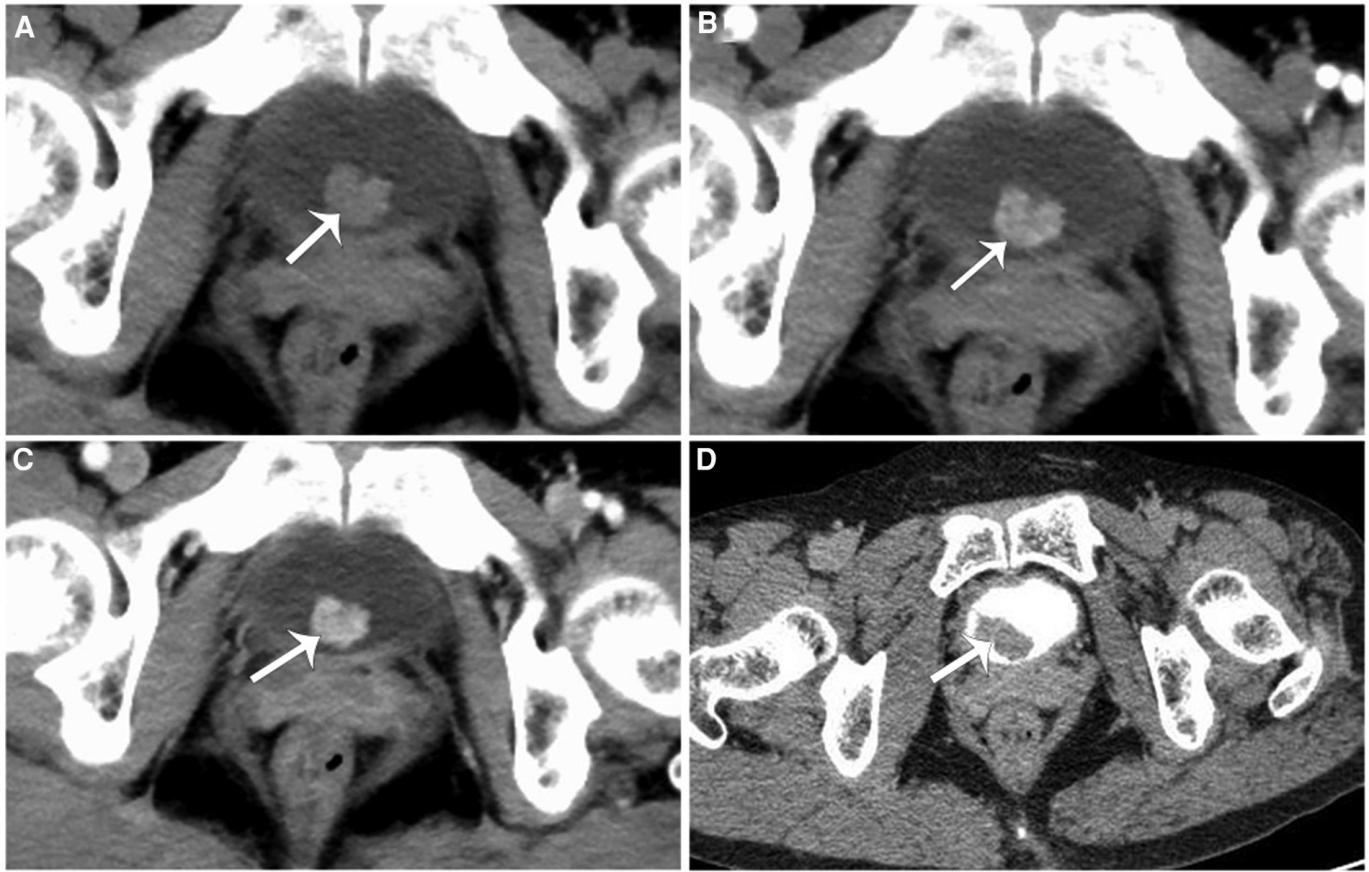
(**A–C**) A cauliflower-shaped soft tissue density nodular shadow (white arrow) was seen in the right posterior lower wall of the bladder, measuring approximately 12 mm × 11 mm × 11 mm, with a wide base attached to the bladder wall and an irregular morphology. (**D**) Enhancement scan, computed tomography urography (CTU) showed a soft tissue density filling defect in the right posterior lower wall of the bladder.

**Figure 2:**
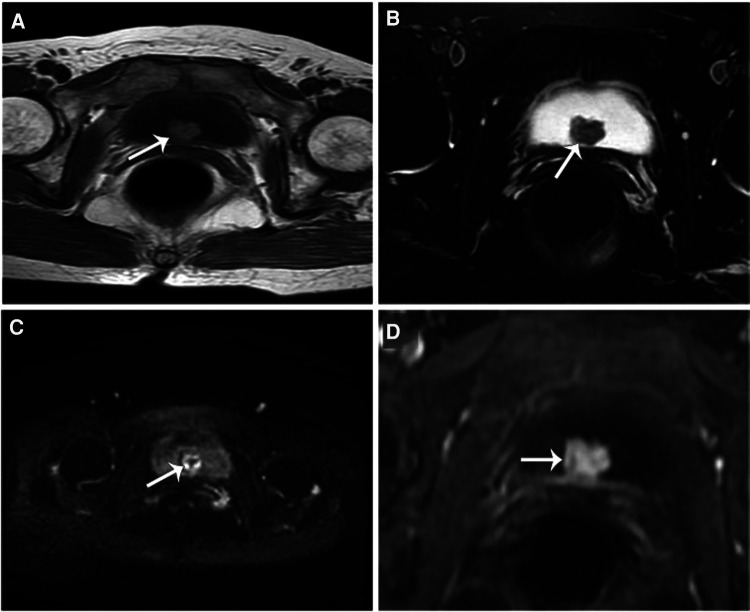
T1wt1 image (**A**) and STIR images (**B**) showed cauliflower-like soft tissue signal nodular shadow (white arrow) in the posterior bladder wall, which was isosignal compared with that of the muscle. It had a wide basal connection with the bladder wall. High signal nodular shadow on DWI (**C**), with significant inhomogeneous enhancement on enhancement scan (**D**).

(1) Laboratory examination: Urine routine: 249 red blood cells/µl, urinary nitrite +, urinary leukocytes +++, fasting blood glucose: 8.05 mmol/L and glycosylated hemoglobin: 7.5%. No malignant cells were seen in urine-based cytology for three consecutive days. Urine culture and drug sensitivity: *Escherichia coli* >10^5^, sensitive to piperacillin and cotrimoxazole, levofloxacin as an intermediary agent.
(2) Imageological examination: ① Computerized Tomography (CT) report: Space- occupying lesions of the bladder, high probability of bladder cancer, multiple stones in both kidneys, cysts in both kidneys, and mild hydronephrosis in the right kidney. A cauliflower-shaped soft tissue density nodular shadow (Figure 1) was seen in the right posterior lower wall of the bladder. ② Magnetic Resonance Imaging (MRI) report: The space-occupying lesions of bladder neck: high probability of bladder cancer (grade:VI-RADS2). The involute papilloma was to be removed. T1WT1 image (Figure 2A) and STIR images (Figure 2B) showed cauliflower-like soft tissue signal nodular shadow (white arrow) in the posterior bladder wall.
(3) Preoperative diagnosis: ① The space-occupying lesions of bladder: bladder cancer? ② Multiple stones in both kidneys; ③ Urinary tract infection; ④ Hypertensive disease grade 3, high-risk group; ⑤ Type 1 diabetes mellitus.

## Therapeutic intervention

**Figure 3:**
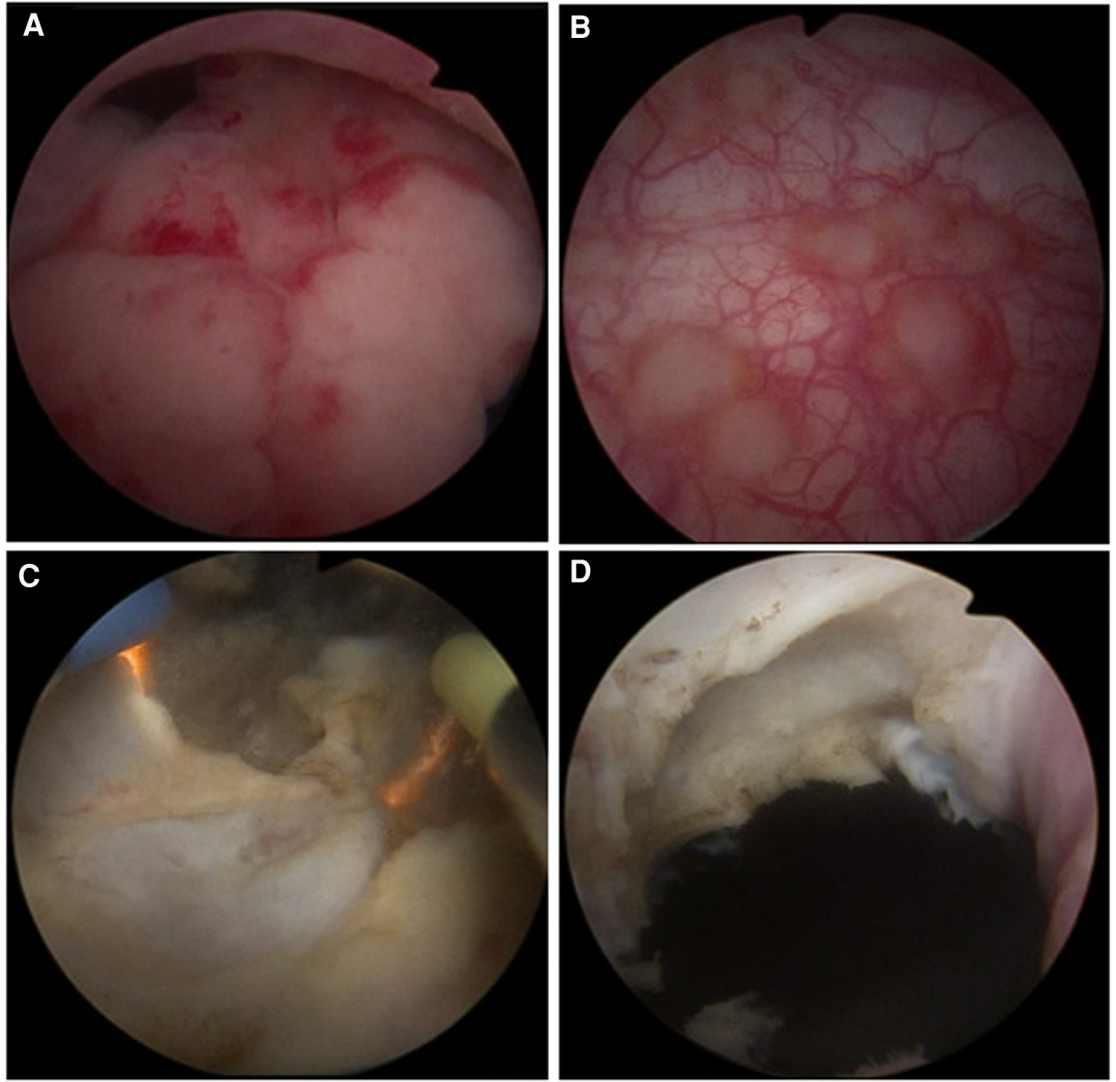
(**A**) A mass with smooth surface mucosa and irregular shape was observed at the internal urethral opening of the bladder neck. (**B**) Extensive multiple superficial mucosal red velvet-like lesions were seen in the bladder and random biopsies were taken. (**C**) Plasma electrode section of the mass with a tough texture and rich blood supply was performed, and all the excised specimens underwent pathological examination. (**D**) Complete excision of the mass reaching the superficial layer of the internal sphincter of the bladder neck.

**Figure 4:**
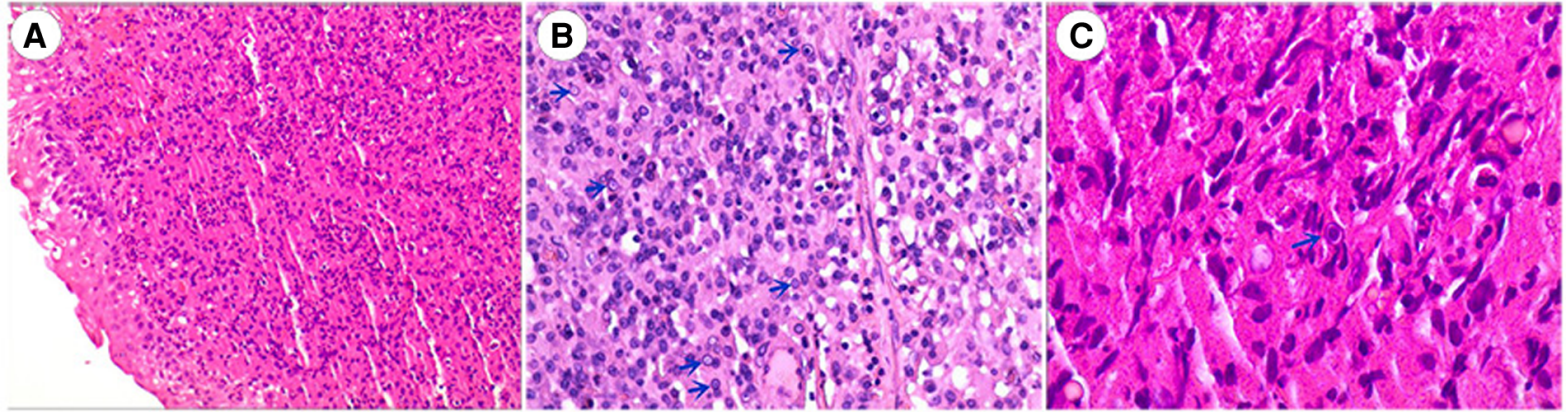
(**A**) HE staining (100×): bladder surface epithelial polarity was present, no significant cellular anisotropy was seen, and submucosal histiocytosis with lymphoplasmacyte-dominated inflammatory cell infiltration was seen. (**B**) HE staining (200×), numerous concentric circular inclusion bodies with Michaelis-Gutmannn vesicles (blue arrows) were seen in histiocytes. (**C**) HE staining (400×), Michaelis-Gutmann vesicles (blue arrow) were magnified.

**Figure 5:**
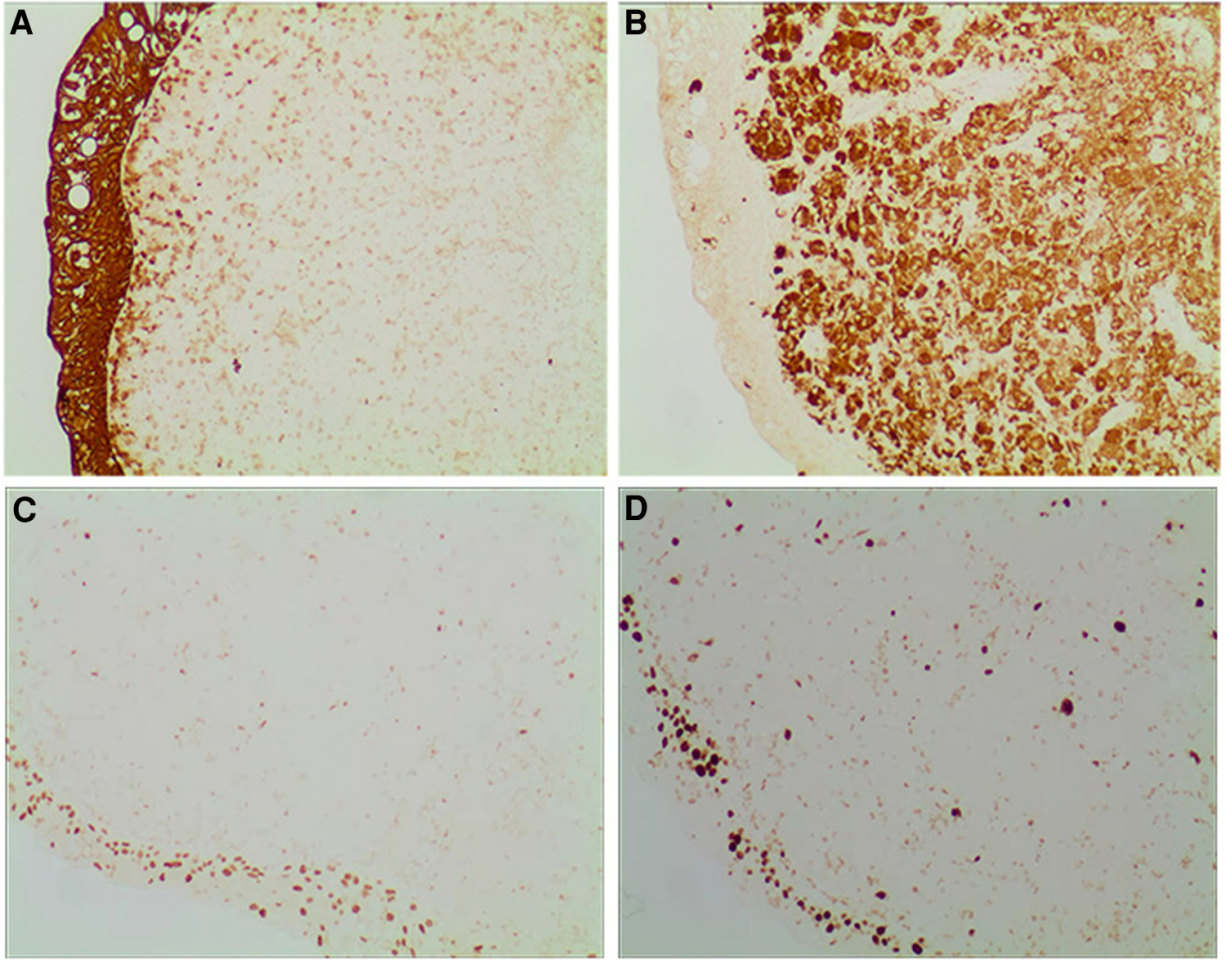
Immunohistochemistry (100×) (**A**) keratoconus protein: broad-spectrum cytokeratin was positively expressed in the overlying uroepithelium and negatively in the interstitial cells. (**B**) CD86: CD68 protein was weakly expressed in the overlying uroepithelium and diffusely expressed in the interstitial cells. (**C**) GATA3: GATA3 protein was diffusely expressed in the interstitial cells and weakly expressed in the overlying uroepithelium. (**D**) Ki-67: Ki-67 protein was diffusely expressed in the interstitial cells and weakly expressed in the overlying uroepithelium.

**Figure 6:**
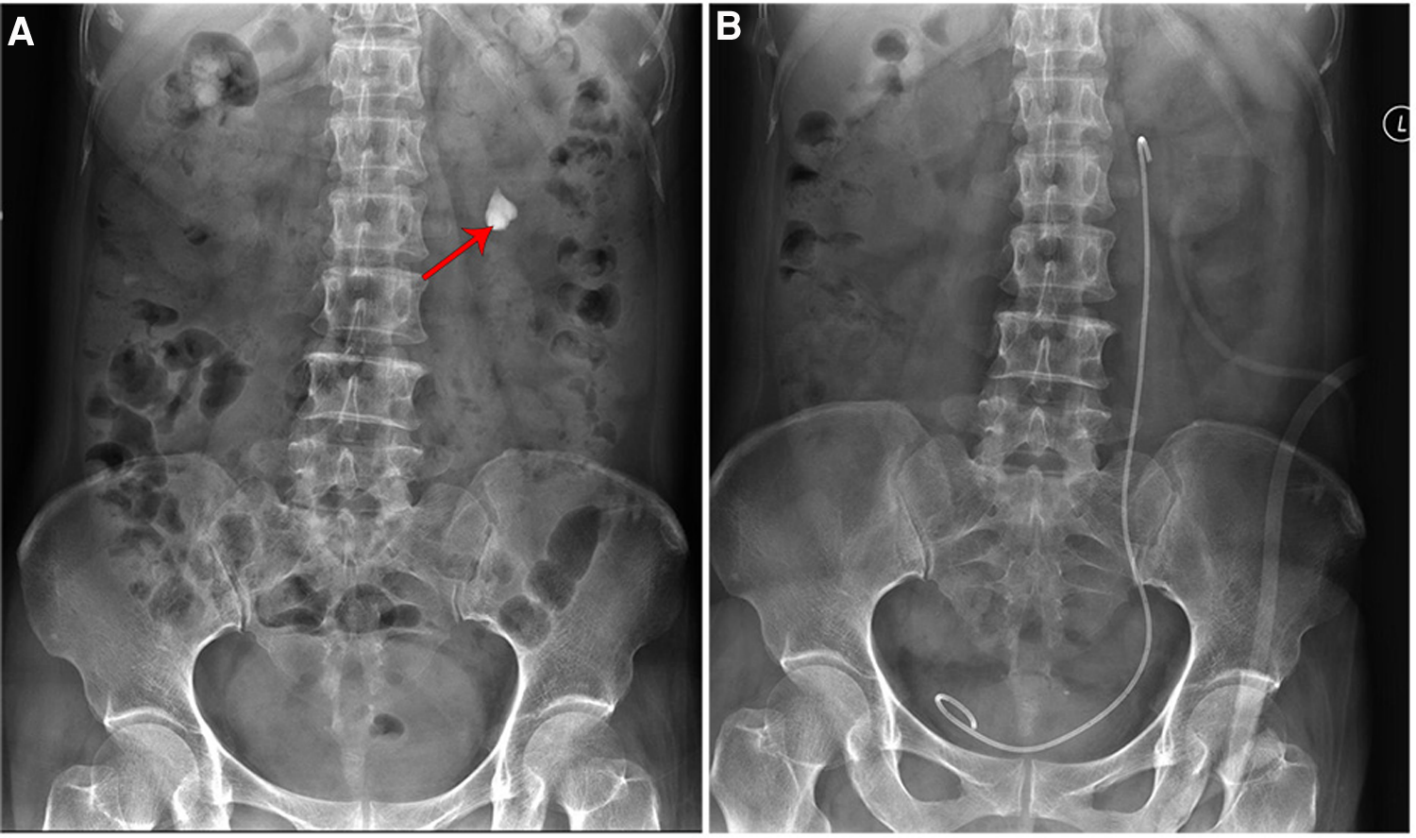
Kidney-ureter-bladder (KUB) showed (**A**) a stone in the left lower renal calyx (red arrow, 20 mm × 17 mm). (**B**) Renal calyx was removed after percutaneous nephrolithotomy.

The patient was given 4.5 piperacillin (IV, q8 h) preoperatively for anti-infection for 1 week, and the recheck revealed that nitrite was converted to negative in the urinary routine. On April 27, 2021, the transurethral bladder mass electrosurgery was conducted under combined spinal and epidural analgesia (with surgical pictures shown in Figure 3), piperacillin anti-infective therapy was continued after operation. Pathological diagnosis of specimen after electrodesiccation: Bladder malacoplakia (Figure 4). Immunohistochemistry (Figure 5): CK-pan (surface epithelial+), GATA-3 (surface epithelial+), CD68 (histiocytes +) Ki67 (+). On May 6, 2021, The ultrasound-guided left percutaneous nephrolithotomy (PCNL) was performed under general anesthesia, and the postoperative kidney-ureter-bladder (KUB) showed that the stone was removed (with KUB pictures shown in Figure 6). Bacterial culture of the extracted left kidney stone indicated a large number of *E. coli*, a small number of fecal alkaline-producing bacilli, and the drug-sensitive results were consistent with the urine culture. Analysis of stone composition revealed calcium oxalate monohydrate, calcium oxalate dihydrate, carbapatite, and ammonium magnesium phosphate hexahydrate (infected stone).

## Follow-up and outcomes

After discharge, the patient was given oral cotrimoxazole (0.96 BID) for 1 month, and the urinary routine was normal on recheck. The Double J ureteral stent was removed 1 month after surgery, and microscopic examination revealed that the multiple spotted bladder lesions were less severe than before. She was advised to take levofloxacin for 1 month. At follow-up to date, the patient had a good prognosis with no discomfort, and cystoscopy showed no bladder mass and erythema lesions of bladder mucosa basically disappeared.

## Discussion

The malacoplakia of the bladder is common in middle-aged adults, with a male to female ratio of 1:4 (4). The main etiologies are bacterial microbial infection, defective immune response (5), and abnormal macrophage function (6). This disease usually occurs in immunocompromised individuals, with *E. coli* being the most common pathogen. The malacoplakia of the bladder combined with yellow granulomatous cystitis has been reported previously (7). Cases of bladder stones in combination with malacoplakia of the bladder have been reported earlier (8), and in this case, the patient had diabetes mellitus and renal stones with recurrent urinary tract infections. Urine culture suggested *E. coli*. Patients with malacoplakia of the bladder present clinically with symptoms of bladder irritation, or with vague pain and hematuria. Bilateral ureteral obstruction as a result of malacoplakia of the bladder (9). In this case, the lesion was located in the inner orifice of the bladder neck urethra, producing a piston-like action, and interruption of urination occurred.

The imaging and cystoscopic presentation of bladder chondromalacia resemble that of a bladder occupying lesion, and at the initial diagnosis, it is mostly considered to be a bladder tumor. Superficial bladder mucosal lesions need to be differentiated from bladder carcinoma *in situ*, adenocystitis, and other diseases. Pathological examination is the gold standard for the diagnosis of this disease. In this case, the clinical diagnosis of bladder cancer was proposed, and the final pathology confirmed the diagnosis, with typical Michaelis-Gutmannn microsomes seen on pathological examination.

Malacoplakia is a relatively rare chronic granulomatous inflammatory disease with a good prognosis but is prone to recurrence. The treatment often varies based on the predilection site, disease severity, and clinical presentation of patients. Currently, there is no standardized treatment available. Regular and effective anti-infective treatment can control the disease, and quinolones are the primary drugs for current treatment, which can improve the survival rate of patients (10) and be used at low doses for a long time to prevent a recurrence (11). For obvious occupancy or when antibiotic treatment is ineffective, the best solution seems to be surgery combined with antibiotic treatment (6). In this case, after preoperative anti-infective treatment, the transurethral electrodesiccation was performed on the bladder mass. Another elective left PCNL was performed to manage the renal stone and aggressively remove infection triggering factors. The patient who adhered to anti-infective treatment after discharge had a good prognosis in the 6-month follow-up. Collectively, this case suggested that thorough treatment of urinary stones and urinary tract infections, together with electrodesiccation of the bladder in possession, which is beneficial in the treatment of malacoplakia of the bladder.

In conclusions, malacoplakia of the bladder and bladder cancer are easily confused in preoperative imaging diagnosis, and easily misdiagnosed as bladder tumor at initial diagnosis, so we must rely on pathological examination to make the final diagnosis. For the treatment of malacoplakia of the bladder, regular anti-infective treatment can be given before surgery, and then combined with PCNL, it can effectively treat the malacoplakia of the bladder.

## Data Availability

The original contributions presented in the study are included in the article/Supplementary Material, further inquiries can be directed to the corresponding author.
